# A case–control study in Taiwanese cohort and meta-analysis of serum ferritin in pancreatic cancer

**DOI:** 10.1038/s41598-021-00650-7

**Published:** 2021-10-28

**Authors:** Ji Min Park, Chen-Zou Mau, Yang-Ching Chen, Yen-Hao Su, Hsin-An Chen, Shih-Yi Huang, Jung-Su Chang, Ching-Feng Chiu

**Affiliations:** 1 School of Nutrition and Health Sciences, College of Nutrition, Taipei Medical University, Taipei, 11031 Taiwan; 2 Graduate Institute of Metabolism and Obesity Sciences, College of Nutrition, Taipei Medical University, Taipei, 11031 Taiwan; 3 Graduate Institute of Clinical Medicine, College of Medicine, Taipei Medical University, Taipei, 11301 Taiwan; 4 Division of General Surgery, Department of Surgery, Shuang Ho Hospital, Taipei Medical University, New Taipei City, 23561 Taiwan; 5 Department of General Surgery, School of Medicine, College of Medicine, Taipei Medical University, Taipei, 11301 Taiwan; 6 Nutrition Research Center, Taipei Medical University Hospital, Taipei, 11031 Taiwan; 7 TMU Research Center of Cancer Translational Medicine, Taipei Medical University, Taipei, 11031 Taiwan

**Keywords:** Biomarkers, Cancer, Gastrointestinal cancer, Tumour biomarkers

## Abstract

Pancreatic cancer is one of the most lethal diseases which lack an early diagnostic marker. We investigated whether serum ferritin (SF) reflects risk for pancreatic cancer and potential genes that may contribute ferritin and pancreatic cancer risks. We performed a meta-analysis of relevant studies on SF and pancreatic cancer risk by searching articles in PUBMED and EMBASE published up to 1 March 2020. We also collected serum samples from Taipei Medical University Joint Biobank and compared SF levels in 34 healthy controls and 34 pancreatic cancer patients. An Oncomine database was applied as a platform to explore a series of genes that exhibited strong associations between ferritin and pancreatic cancer. Herein, we show that high levels of SF can indicate risk of pancreatic cancer, suggesting SF as the new tumor marker that may be used to help pancreatic cancer diagnosis. We also found that expressions of iron homeostasis genes (*MYC*, *FXN*) and ferroptosis genes (*ALOX15*, *CBS*, *FDFT1*, *LPCAT3*, *RPL8*, *TP53*, *TTC35*) are significantly altered with pancreatic tumor grades, which may contribute to differential expression of ferritin related to pancreatic cancer prognosis.

## Introduction

Pancreatic cancer is the fourth leading cause of cancer-related deaths in the US, and the number of deaths also continues to increase in Taiwan^1,2^. The age-standardized mortality rate for pancreatic cancer in Taiwan is 5.6 per 100,000 people, and it was ranked the seventh leading cause of mortality in 2018^3^. It is reported that approximately 80% of pancreatic cancer patients present an unresectable tumor with distant metastasis at the time of diagnosis, due in part to the absence of an early diagnostic test^4^. As a result, the 5-year survival rate of pancreatic cancer was 9% in 2020, in which the pattern of cancer deaths closely paralleled that of the incidence^1,5^. Considering the poor prognoses and limited therapeutic options, there is an urgent need to develop potential biomarkers for the early diagnosis of pancreatic cancer.

Iron is an essential element for most forms of life due to its roles in synthesizing oxygen transport proteins such as hemoglobin, myoglobin, and other iron-containing proteins. It is widely acknowledged that an excess accumulation of iron can be harmful by increasing production of reactive oxygen species (ROS), which ultimately cause DNA damage and genetic alterations, leading to tumor growth^6–9^. Ferritin is the primary iron storage protein, and its serum level reflects iron stores in the body. It was reported that 1 ng/mL serum ferritin (SF) reflects approximately 8 mg of stored iron^10–12^. As high levels of iron are potentially toxic, several epidemiological studies also found associations between high levels of SF and increased risks for various cancers, suggesting that SF could be used as an iron index and also a tumor marker^13–16^. Degradation of ferritin, on the other hand, can elevate the level of cellular labile iron, which increases ROS levels and ultimately induces an iron-dependent form of non-apoptotic cell death, called ferroptosis^17^. Ferritin, thus, is an important mediator that regulates cellular iron metabolism and carcinogenesis, which may ultimately provide new insights into cancer research and therapeutic strategies.

Correspondingly, SF was suggested to be potential biomarker for diagnosing several types of cancer or monitoring cancer progression. Recent studies also suggested a plausible link between SF and pancreatic cancer, but those findings are not yet definitive. The present study was designed to explore if SF is associated with pancreatic cancer. We specifically assessed associations between SF and pancreatic cancer risk in Taiwan, by analyzing ferritin assays in 34 Taiwanese patients with histologically proven pancreatic carcinoma and in 34 healthy Taiwanese controls. We also performed a pooled analysis of six studies for a comprehensive quantitative assessment of the association of SF levels with pancreatic cancer risks. Lastly, an Oncomine database analysis of patient-derived gene expressions was used to assess potential genes that might mediate the association between ferritin and pancreatic cancer.

Combined together, in response to pancreatic cancer is being considered one of the most lethal malignancies with an absence of an early diagnostic test, our study investigated the significance of SF as a prognostic marker for pancreatic cancer and offers new possibilities for pancreatic cancer research to improve overall prognoses. SF levels could serve as a potential marker to predict individuals at increased risk during pancreatic cancer screening, along with being a novel therapeutic strategy for pancreatic cancer.

## Results

### Meta-analysis of serum ferritin and pancreatic cancer risks

**Figure 1:**
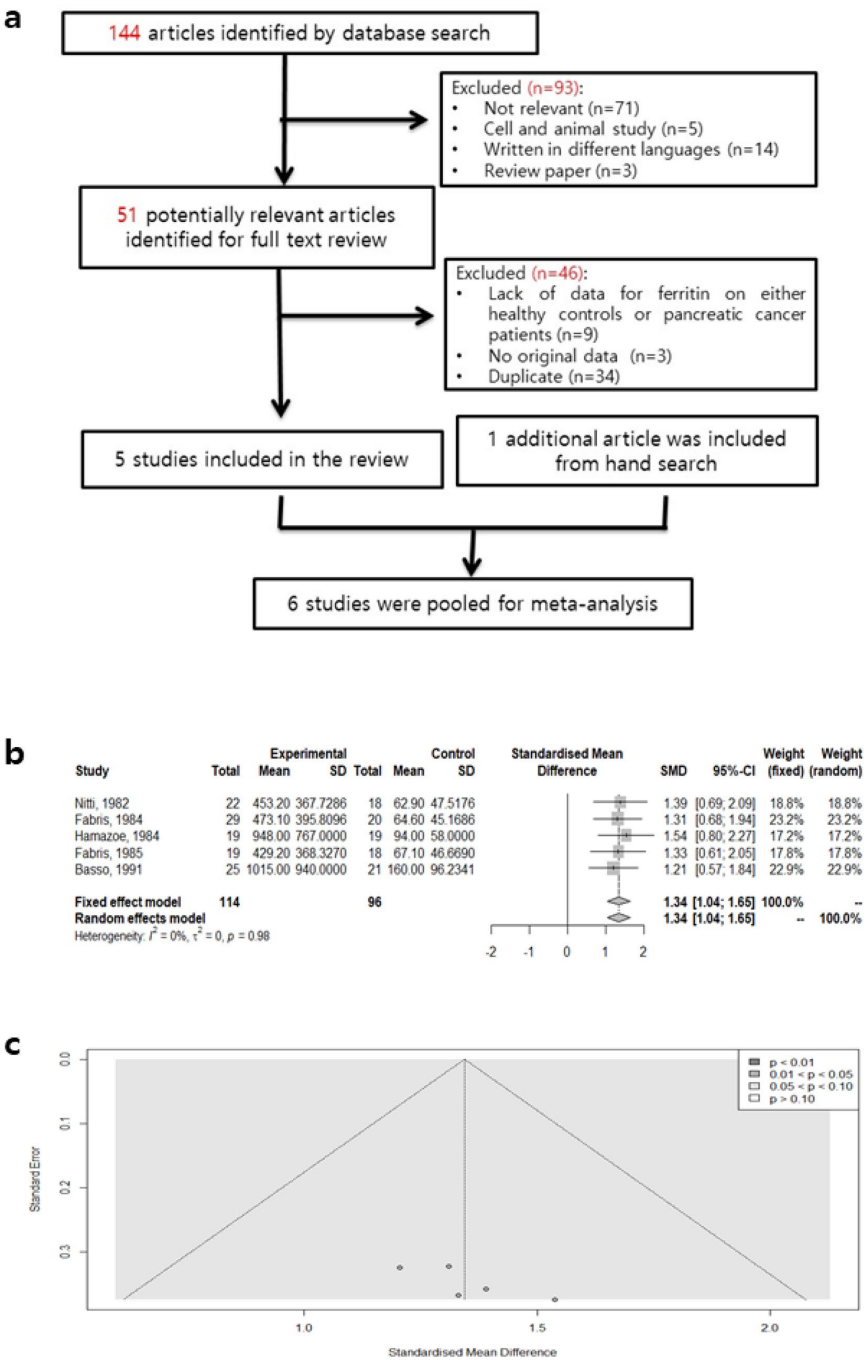
Meta-analysis of SF and pancreatic cancer risks. (**a**) Flow diagram of screened and included papers for meta-analysis. (**b**) Forest plot of studies in serum ferritin for subjects with pancreatic cancer (Experimental; n = 114) versus healthy controls (Control; n = 96). Standard mean difference (SMD) and 95% confidence intervals (CI) were calculated on the basis of both fixed-effect and random-effect models. (**c**) Funnel plot of studies in serum ferritin for subjects with pancreatic cancer versus healthy controls.

To summarize previous studies investigating the association between SF and pancreatic cancer risks, we conducted a meta-analysis of individual datasets. In total, 144 articles were searched from PUBMED and EMBASE, among all articles published up to 1 March 2020. After screening the title and abstract, 51 articles were retrieved and reviewed, of which five articles met the inclusion criteria. An additional article was included from a hand search (Fig. 1a). In total, six studies that included 119 pancreatic cancer patients and 196 healthy controls were collected, comprising four articles from Europe (Italy) and two articles from Asia (China and Japan). In these studies, SF was measured by an immunoradiometric assay (IRMA), 2-site IRMA, or radioimmunoassay (RIA) as summarized in Table 1.

**Table 1:** Summary of case–control studies investigating levels of serum ferritin in pancreatic cancer patients.

Study first author	Country	Measurement	Healthy controls				Pancreatic cancer				
			Age (years)	N	% of women	Concentration, ng/ml (mean ± SD)	Age (years)	N	% of women	Concentration, ng/ml (mean ± SD)	Stage of the disease
Nitti^18^	Italy	IRMA	23–39	18	28	62.9 ± 47.5	41–70	22	45	453.2 ± 367.7	NA
Fabris^19^	Italy	IRMA	23–39	20	30	64.6 ± 45.2	41–70	29	38	473.1 ± 395.8	NA
Hamazoe^20^	Japan	RIA	NA	19	0	94 ± 58	NA	19	NA	948 ± 767	NA
Chen^21^	China	RIA	NA	100	NA	129 ± 57.6	NA	5	NA	573.4 ± 251.1	NA
Fabris^22^	Italy	IRMA	24–39	18	28	67.1 ± 46.7	45–70	19	53	429.2 ± 368.3	(n = 2) T2N1M1 (n = 4) T3NOMO (n = 1) T3N1MO (n = 12) T3N1M1
Basso^23^	Italy	2-site IRMA	24–61	21	38	160 ± 96.2	43–73	25	40	1015 ± 940	NA

*IRMA* immunoradiometric assay, *RIA* radioimmunoassay, *NA* not announced.

We observed elevated SF expression in pancreatic cancer patients compared to the controls (summary SMD = 2.01, 95% CI = 1.05–2.97, Z value = 4.15, *p* for the Z test < 0.0001) with extreme heterogeneity (I^2^ = 90.4%) and publication bias (*p* = 0.002; Supplementary Figs. S1–2). However, high heterogeneity was observed in the analysis, which was mainly attributable to a study by Chen et al.^21^ Meta-regression models were conducted to explore potential heterogeneity that may have impacted the association between SF and pancreatic cancer. We found that number of controls (I^2^ = 0.0%, *p* < 0.001) and patients (I^2^ = 82.2%, *p* < 0.001) were the possible sources of heterogeneity (Supplementary Table S2). When we excluded this study, the association between SF and pancreatic cancer was still statistically significant (Fig. 1b; summary SMD = 1.34, 95% CI = 1.04–1.65, Z value = 8.66, *p* for the Z test < 0.0001), and no heterogeneity was found (I^2^ = 0.0%). Indeed, only the study from Fabris et al.^22^ reported the stage of pancreatic cancer patients, but no subgroup analysis of SF and cancer stage was performed (Table 1). Publication bias was measured using funnel plots and Egger’s test (*p* = 0.112), the results of which indicated that no publication bias existed (Fig. 1c). The results indicated no source of heterogeneity in the association between SF levels and pancreatic cancer risk (Supplementary Table S3).

### Serum ferritin and pancreatic cancer risks in Taiwan

**Figure 2:**
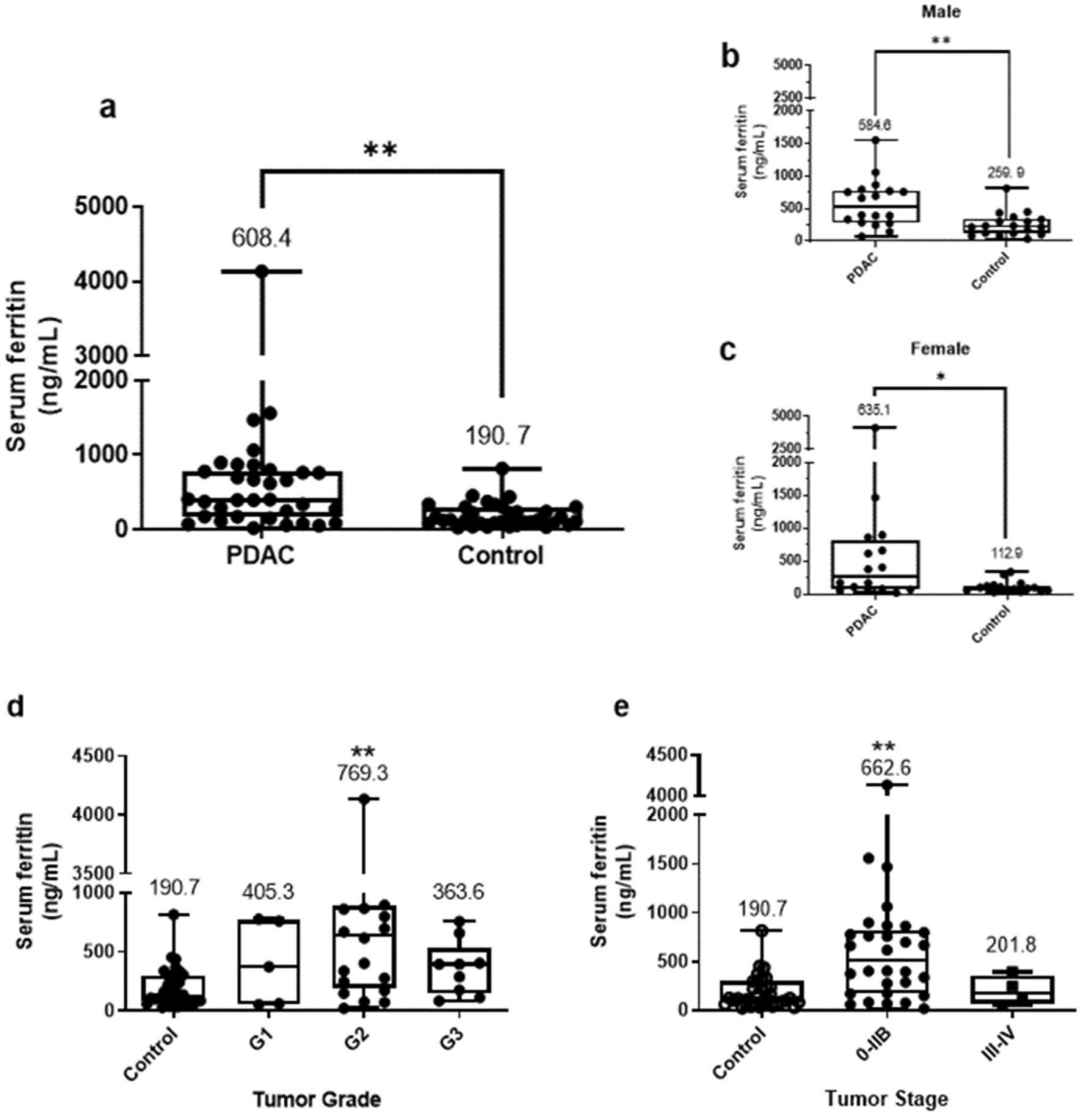
Association of serum ferritin (SF) with pancreatic cancer risks in Taiwan. (**a**) Levels of SF in pancreatic ductal adenocarcinoma (PDAC) patients (n = 34) and healthy controls (n = 34). (**b**, **c**) Serum levels of ferritin sub-grouped by gender. *p < 0.05 and **p < 0.01 compared to the controls as determined by Student’s *t*-test. SF for subjects with healthy controls and pancreatic cancer patients was classified according to their (**d**) histological grade: G1 (n = 5), G2 (n = 20), and G3 (n = 9), (**e**) tumor stage: stage 0–IIB (n = 30) and stage III–IV (n = 4). **p < 0.01 compared to the controls as determined by one-way ANOVA with Tukey’s post-hoc tests.

In this case–control study, we analyzed SF levels in 68 Taiwanese participants, comprising 34 (50%) PDAC patients aged 41–82 years and 34 (50%) healthy controls aged 57–74 years, to investigate the relationship between SF and pancreatic cancer (Supplementary Table S4). Results revealed that there was no statistically significant difference between the two groups with respect to sex or age, but a higher mean concentration of SF was detected in PDAC patients (608.4 ± 126.3 ng/ml) compared to that in healthy controls (190.7 ± 28.1 ng/ml). The difference in SF levels between two groups was statistically significant, as shown in Fig. 2a (*p* < 0.01), and the same pattern existed when we sub-grouped subjects by gender (Fig. 2b, c). Next, we examined whether SF was an independent predictor of histologic severity in Taiwanese PDAC patients. We sub-grouped PDAC patients based on the histologic grade and analyzed SF levels in each group (Supplementary Table S5). Results revealed that the SF concentration was highest in the G2 group with a mean value of 769.3 ± 203.2 ng/ml, followed by the G1 group with 405.3 ± 159.5 ng/ml, and the G3 group with 363.6 ± 77.38 ng/ml. Each group of patients with different histological tumor grades also showed statistically significant differences in SF compared to that of the controls, and the difference was the highest in the G2 group compared to the G1 and G3 groups (Fig. 2d). We also sub-grouped Taiwanese pancreatic cancer patients based on the cancer stage and analyzed the SF level in each group (Supplementary Table S5). PDAC patients at stage 0-IIB showed significant increase compared to the controls (*p* = 0.0019) but no significant difference was found in SF level of patients at stage III–IV (Fig. 2e).

### Ferritin-associated genes and pancreatic cancer risks

#### Ferritin subunits: heavy chain and light chain

**Figure 3:**
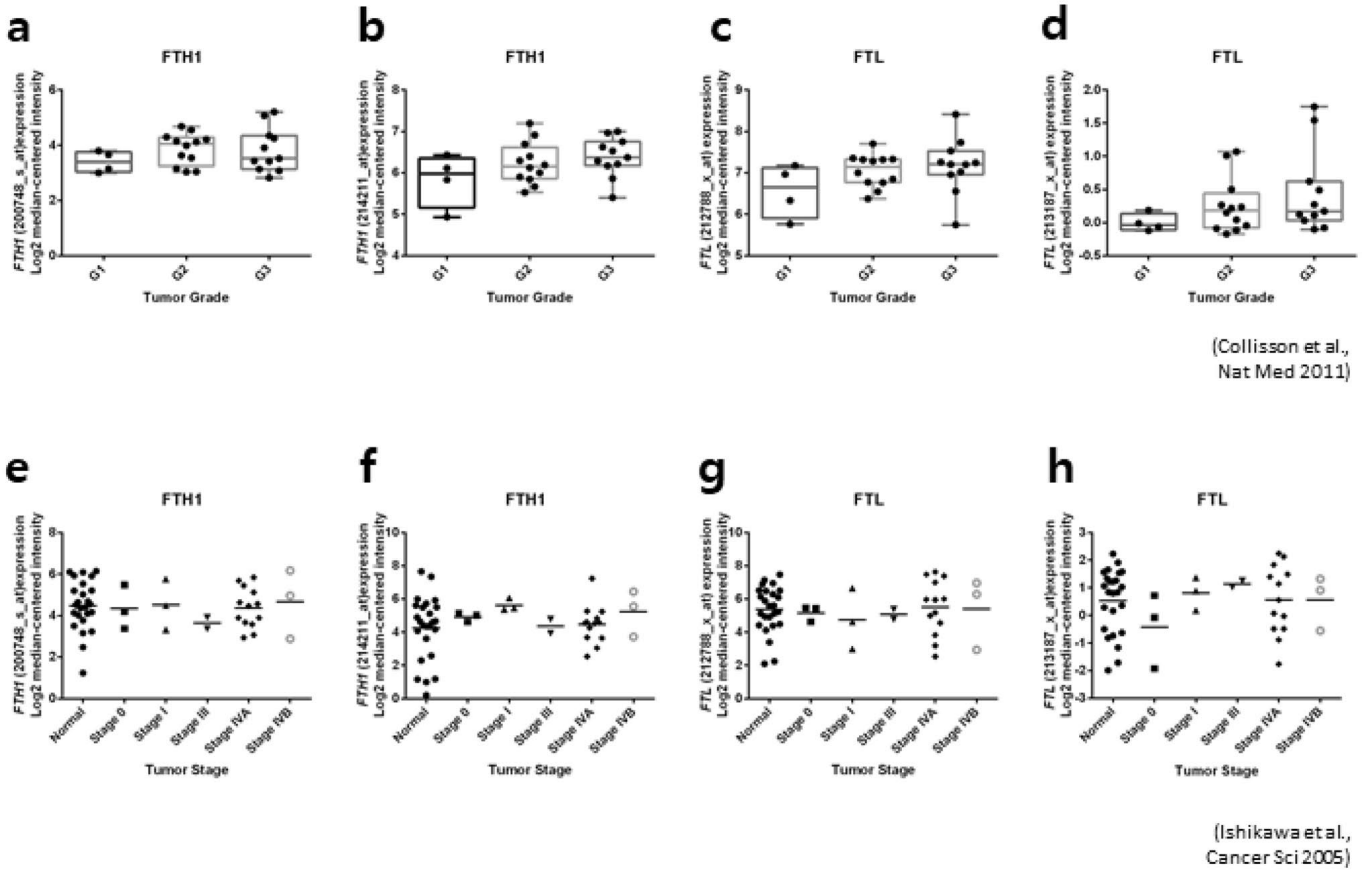
Oncomine analysis of Ferritin high chain (*FTH1*) and ferritin light chain (*FTL*) mRNA expressions in normal and/or human pancreatic cancer tissues. (**a**–**d**) *FTH1* and *FTL* mRNA expression profiles in different cancer grades: G1 (n = 4), G2 (n = 12), G3 (n = 11) and (**e**–**h**) stages: normal subjects (n = 25), stage 0 (n = 3), stage I (n = 3), stage III (n = 2), stage IVA (n = 13), and stage IVB (n = 3) pancreatic cancer patients.

Ferritin is composed of two subunit types, with ferritin high polypeptide 1 (FTH1) participating in ferroxidase activity and being devoted to rapid uptake and release, while ferritin light polypeptide (FTL) functions in the long-term storage of iron^24^. As a precise reason for the involvement of ferritin in pancreatic carcinogenesis has not yet been fully determined, previous study reported that SF may derived from hepatocytes, macrophages, and microglia and contains both FTH1 and FTL ferritin subunits^25^. It is also reported that SF in malignant histiocytosis is mainly composed of FTH1, whereas SF in breast cancer patients is highly correlated with FTL^26–28^. We thus examined messenger (m)RNA expressions of *FTH1* and *FTL* in pancreatic cancer patients using Oncomine datasets. We analyzed a dataset from Collisson et al.^29^ which included 27 pancreatic cancer tissues of patients with histological grades G1, G2, and G3. As shown in Fig. 3a–d, there were strong trends of increased expressions in both *FTH1* and *FTL* with higher-grade tumors, but they did not reach statistical significance. Additionally, we analyzed a dataset from Ishikawa et al.^30^ which included 49 sample tissues with 25 normal and pancreatic cancer patients at stages 0 (n = 3), I (n = 3), III (n = 2), IVA (n = 13), and IVB (n = 3). mRNA expressions of neither *FTH1* nor *FTL* were significantly associated with clinical stages of pancreatic cancer (Fig. 3e–h).

#### Iron homeostasis genes

**Figure 4:**
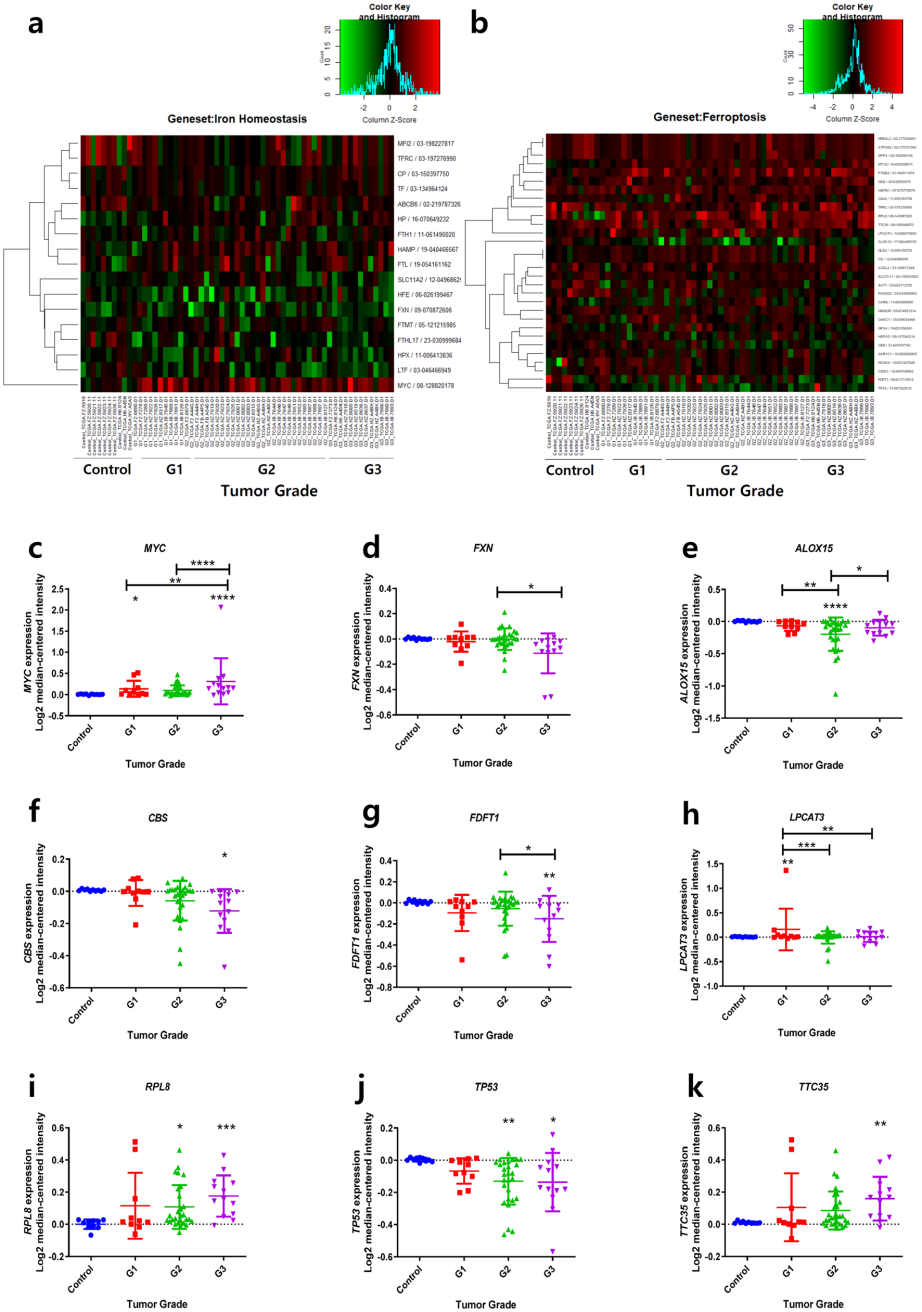
Analysis of pancreatic cancer patient gene expression data responsible either iron homeostasis signaling or ferroptosis. Heatmaps for gene expressions of (**a**) iron homeostasis signaling and (**b**) ferroptosis was created using R Studio 3.5.3.^33^ using the package of heatmap.plus (ver. 1.3)^34^ on TCGA pancreatic cancer dataset. Differential gene expressions in 10 normal tissues and 51 tumor tissue samples at different histological grades (G1: n = 10, G2: n = 28, G3: n = 13) are calculated based on the Z-score showing an up-regulation (red) and down-regulation (green) clustered in heatmaps. Columns represent each sample. (**c**, **d**) Iron homeostasis genes and (**e**–**k**), ferroptosis regulatory genes were differentially expressed with histologic tumor grade of pancreatic cancer. Only genes that reached statistical significance are reported. *p < 0.05, **p < 0.01, ***p < 0.001, and ****p < 0.0001 as determined by a two-way ANOVA with Tukey’s post-hoc tests.

Ferritin, as a cellular iron storage protein, is an important regulator in maintaining iron homeostasis; therefore, in most cases, an increased SF level indicates high amounts of iron stores in the body^31,32^. A gene expression analysis, analyzing 10 normal tissues and 51 pancreatic cancer tissues from TCGA datasets, was performed to investigate whether iron homeostasis genes contributed to the association between ferritin and pancreatic cancer risks. We first used a heatmap analysis to visualize key iron metabolism genes, the expressions of which correlated with the histological grades (control and G1–G3) of pancreatic cancer (Fig. 4a). Results revealed that low expression of *FXN* (frataxin) and high expression of *MYC* were associated with advanced grades of pancreatic tumors (Fig. 4c, d). In particular, *MYC* gene expression was significantly higher in G3 pancreatic tumors compared to any other grades of pancreatic cancer, suggesting that elevated levels of *MYC* may contribute to the mechanisms underlying ferritin and increased risks of pancreatic cancer.

#### Ferroptosis regulator genes

Ferritin has an important role in mediating ferroptosis in cancer cells^35^. We also used a heatmap to visualize ferroptosis regulatory genes, the expressions of which were correlated with histological grades (control and G1–G3) of pancreatic cancer (Fig. 4b). Results revealed that the key genes associated with pancreatic cancer were *ALOX15* (arachidonate 15-lipoxygenase), *CBS* (cystathionine-β-synthase), *FDFT1* (farnesyl diphosphate farnesyl transferase 1), *LPCAT3* (lysophosphatidylcholine acyltransferase 3), *RPL8* (ribosomal protein L8), *TP53*, and *TTC35* (tetratricopeptide repeat domain 35), the expressions of which statistically significantly differed between normal tissues and pancreatic tumors of higher histological grades (Fig. 4e–k).

#### Correlation between ferritin, iron homeostasis and ferroptosis regulator genes

We conducted a correlation analysis to investigate whether iron homeostasis genes, *FXN* and *MYC*, are also associated with ferritin in contributing pancreatic cancer progression (Table 2). From a correlation analysis of TCGA dataset, we found that both *FXN* and *MYC* were positively correlated with *FTH1* (r = 0.654; *p* = 0.0402), whereas *MYC* was positively correlated with *FTL* (r = 0.662; *p* = 0.0369) in the control group. In any grade of pancreatic tumor tissues, however, no statistically significant correlation was found between either *FTH1* or *FTL* and an iron homeostasis gene set. Although more data are needed to confirm the association, our result suggests that *FTH1* and *FTL* may be involved in iron homeostasis for maintaining normal intracellular iron levels in cells.

**Table 2:** Analysis of correlations of iron metabolism and ferroptosis genes with ferritin subunits, *FTH1* and *FTL*.

Genes	*FTH1*								*FTL*							
	Control		G1		G2		G3		Control		G1		G2		G3	
	r	*p*	r	*p*	r	*p*	r	*p*	r	*p*	r	*p*	r	*p*	r	*p*
*FXN*	0.654	0.0402*	− 0.608	0.0625	0.230	0.2388	− 0.514	0.0727	0.383	0.2753	− 0.422	0.2245	− 0.0247	0.9006	− 0.402	0.1739
*MYC*	0.639	0.0469*	− 0.551	0.0990	0.0599	0.7620	0.081	0.7922	0.662	0.0369*	0.607	0.0628	0.313	0.1052	− 0.229	0.4509
*ALOX15*	0.606	0.0631	0.526	0.1184	− 0.186	0.3446	− 0.0310	0.9202	0.893	0.0005*	− 0.747	0.0131*	− 0.0493	0.8035	0.537	0.0584
*CBS*	0.718	0.0195*	− 0.437	0.2063	− 0.489	0.0083**	− 0.430	0.1424	0.332	0.3489	− 0.522	0.1216	− 0.238	0.2226	− 0.689	0.0092*
*FDFT1*	0.450	0.1921	0.910	0.0003*	0.353	0.0655	− 0.536	0.0589	0.761	0.0106*	− 0.187	0.6053	− 0.272	0.8908	− 0.595	0.0321*
*LPCAT3*	0.751	0.0124*	− 0.979	< 0.001*	− 0.280	0.1495	− 0.192	0.5308	0.544	0.1038	0.127	0.7263	− 0.401	0.8394	− 0.100	0.7441
*RPL8*	0.505	0.1368	− 0.566	0.0884	0.00515	0.9793	0.422	0.1506	0.689	0.0274*	0.447	0.1950	0.206	0.2921	− 0.0378	0.9025
*TP53*	0.606	0.0631	0.520	0.1238	− 0.379	0.0469*	− 0.691	0.0089*	0.893	0.0005*	− 0.738	0.0148*	− 0.223	0.2542	− 0.373	0.2093
*TTC35*	0.671	0.0337*	− 0.567	0.0874	0.112	0.5691	0.376	0.2059	0.310	0.3835	0.441	0.2018	0.381	0.0457*	0.304	0.3128

Correlation of iron metabolism and ferroptosis genes with ferritin subunits, *FTH1* and *FTL* expression was determined by the Pearson correlation coefficient (r).

Statistical significance is presented in table: *p < 0.05.

Suppression of FTH1 was also shown to promote ferroptotic cell death in hepatocellular carcinoma cells in response to classic ferroptosis inducers, such as erastin and sorafenib^36^. Hence, we also determined whether the *FTH1* or *FTL* gene was correlated with a set of ferroptosis genes that were revealed to be highly associated with increased risks for pancreatic cancer. The correlation analysis from TCGA dataset showed that *FTH1* displayed significant positive correlations with the *CBS* (r = 0.718; *p* = 0.0195), *LPCAT3* (r = 0.751; *p* = 0.0124), and *TTC35* (r = 0.671; *p* = 0.0337) genes in the control group, while it was strongly negatively correlated with the *CBS* (G2; r = − 0.489; *p* = 0.0083), *LPCAT3* (G1; r = − 0.979; *p* < 0.0001), and *TP53* (G2; r = − 0.379; *p* = 0.0469 and G3; r = − 0.691; *p* = 0.0089) genes in pancreatic tumor tissues (Table 2). *FTL*, on the other hand, displayed significant positive correlations with *ALOX15* (r = 0.893; *p* = 0.0005), *FDFT1* (r = 0.761; *p* = 0.0106), RPL8 (r = 0.689; *p* = 0.0274), and *TP53* (r = 0.893; *p* = 0.0005) in the control group, whereas it was strongly negatively correlated with *ALOX15* (G1; r = − 0.747; *p* = 0.131), *CBS* (G3; r = − 0.689; *p* = 0.0092), *FDFT1* (G3; r = − 0.595; *p* = 0.0321), *TP53* (G1; r = − 0.738; *p* = 0.0148) and *TTC35* (G2; r = − 0.381; *p* = 0.0457) in pancreatic tumor tissues (Table 2). These results indicated that correlations of gene expressions of *FTH1* or *FTL* with the majority of ferroptotic genes in a set were reversed with pancreatic tumors, suggesting that *FTH1* and *FTL* ferritin subunits may interact with key ferroptosis regulators to mediate ferroptosis in pancreatic cancer patients.

## Discussion

Serum level of ferritin has been regarded as the most reliable and convenient laboratory tool for estimating iron stores in the body, which of its concentration reflects the total body iron stores^10,11,37^. To date, several epidemiological studies have linked SF levels with a risk for pancreatic cancer, but the association between SF and pancreatic cancer has not yet yielded conclusive results. In the present study, we performed meta-analysis and a case–control study to summarize the effects of SF levels and pancreatic cancer risks. We found that high SF was associated with increasing risks of pancreatic cancer; thus, SF has potential application as a clinical biomarker in the diagnosis of pancreatic cancer. Furthermore, our study suggested candidate genes that may relate to association between ferritin and pancreatic cancer risks, proposing the potential molecular mechanism of ferritin related to pancreatic cancer.

The reference ranges of normal SF are 30–300 ng/ml for men and 10–200 ng/ml for women, but no cut-off values for SF as an indicator of pancreatic cancer^38^. In this study, we identified that the mean SF concentrations in healthy Taiwanese controls were 259.9 ng/ml in males and 112.9 ng/ml in females, both of which are within the normal range reported by others^38^. Compared to healthy controls, Taiwanese pancreatic cancer patients had significantly higher levels of mean SF, whose levels were 584.6 ng/ml in males and 635.1 ng/ml in females (Fig. 2). These results are consistent with findings from our pooled analysis of six case–control studies, in which the mean SF level of pancreatic cancer patients were all above 400 ng/ml and significantly elevated compared to that of healthy controls (Supplementary Fig. S1). We found that inclusion of a study by Chen et al. mainly contributed to the high heterogeneity, probably due to the small number of pancreatic cancer patients (n = 5) compared to that of healthy controls (n = 100; Supplementary Table S2). Considering that high heterogeneity represents a risk of bias, we excluded that study by Chen et al. and still found that the correlation between expression levels of SF and pancreatic cancer existed without publication bias (Fig. 1b, *p* > 0.05) and heterogeneity (Supplementary Table S3). The meta-analysis, therefore, supported a significant association between SF and pancreatic cancer, in line with the association found in our population-based case–control study in Taiwan.

The pathological assessment of pancreatic cancer is important not only for assessing treatment effects but also for predicting prognostic outcomes. We thus examined whether SF is an independent predictor of histologic severity in Taiwanese pancreatic cancer patients. Histological grades of pancreatic cancer are coded as to how cancerous tissues look similar to normal tissues under the microscope: G1, well-differentiated; G2, moderately differentiated; G3, poorly differentiated; and G4, undifferentiated^39^. It was reported that the tumor grade is highly correlated with adverse outcomes and overall 5-year survival in pancreatic cancer patients^39^. Pancreatic tumor stages, on the other hands, indicate the tumor size, existence in lymph nodes, and the metastasis status, and later stages indicate that the cancer has spread to other organs and, therefore, carries a poor prognosis^39^. Our results revealed that SF is statistically significantly increased in tumor grade 2 and stage 0-IIB pancreatic cancer patients compared to those of the healthy controls (Fig. 2d, e). The small sample sizes in this study may account for the negative results; therefore, additional studies are needed to confirm the effect of SF levels in diagnosing histologic severity.

The exact underlying mechanisms for the detrimental effects of ferritin on pancreatic cancer remain unknown; however, some studies proposed that the role of ferritin in regulating iron homeostasis may be associated with increasing risks of pancreatic cancer^40–42^. It is generally agreed that excess iron accumulation increases oxidative stress by the Fenton reaction and produces hydroxyl radicals, which ultimately damage cells and induce carcinogenesis^43^. Different levels of ferritin are also important in mediating ferroptosis in cancer cells. Although the exact mechanism of which cellular iron species facilitates ferroptosis remains unclear, a recent study reported that an excess of intracellular iron released from degraded ferritin could promote the accumulation of cellular ROS and ultimately lead to ferroptotic cell death in acute promyelocytic leukemia (AML) cells^35^. Thus, we retrieved data from Oncomine and TCGA dataset to identify iron homeostasis and ferroptosis genes that may mediate the association between ferritin and increasing pancreatic cancer risks (Fig. 4, Table 2). Notably, iron homeostasis pathway genes with altered expressions in pancreatic cancer tissues included *FXN* and *MYC*, while ferroptosis pathway genes with altered expressions included *ALOX15, CBS, FDFT1, LPCAT3, RPL5, TP53*, and *TTC35*. We found that iron metabolism genes, low expression of *FXN*, and high expression of *MYC* were associated with increasing risks of pancreatic cancer. *MYC* is known as a proto-oncogene for which elevated activity was found in many cancer types^44^. Regarding iron homeostasis regulation, MYC was shown to suppress FTH1 and stimulate IRP2 (iron regulatory protein-2) expressions, which ultimately increases the intracellular iron pool^45^. Our study, however, revealed that high expression of the *MYC* gene was linked to an increased risk of pancreatic cancer, but it was not significantly correlated with either the *FTH1* or *FTL* gene in different tumor grades, implying that MYC may be involved in regulating iron hemostasis in pancreatic cancer but not through modulating ferritin activity.

Several ferroptosis genes were closely correlated with advanced tumor grades, and their expressions were most significantly altered in G3 pancreatic cancer. There was a trend of decreasing *CBS* and *TP53* gene levels with advanced grades of pancreatic cancer, although statistical significance was shown in either G2 and/or G3 of pancreatic cancer compared to the controls. *CBS* is a gene that mediates ferroptosis inhibition by regulating the trans-sulfuration pathway, which rescues cells from erastin-induced ferroptosis^46^. Surprisingly, we found that *CBS* expression was significantly low in G3 compared to the controls. Moreover, a correlation analysis of *CBS* and *FTH1* showed a significant positive correlation in the controls but a negative correlation in G2 pancreatic tumors. These findings suggest that CBS may stimulate the ferroptotic pathway in pancreatic cancer cells through different mechanisms, possibly involving an interplay between CBS and FTH1. A tumor suppressor gene, *TP53*, was also notably downregulated in G2 and G3 pancreatic tumors, whereas significant negative regulation of the *FTH1* and *FTL* genes was found in different histological grades. A previous study also suggested crosstalk between TP53 and iron regulators, in which anticancer effects of iron chelators, such as deferoxamine and triapine, upon iron depletion are mediated in part by suppressing TP53 activation^47^. It was also reported that TP53 is recruited on the FTH1 promoter by NF-Y which subsequently represses ferritin expression^48^. A recent study suggested that ferroptosis is a major component of TP53 in mediating tumor suppression^47^. Our study demonstrated that the *TP53* level in pancreatic cancer was highly associated with *FTH1* and *FTL*, suggesting that TP53 cooperates with these genes in modulating pancreatic carcinoma. As TP53 appears to modulate both iron metabolism and ferroptosis, a comprehensive understanding of TP53 in modulating ferroptosis needs to be further elucidated, which is required to target pancreatic cancer as a therapeutic strategy.

Both *RLP8* and *TTC35* are known as mitochondrial genes that were shown to suppress erastin-induced ferroptosis^49,50^. Our study found that *RLP8* and *TTC35* genes were significantly increased in advanced grades of pancreatic tumor tissues compared to the controls, suggesting that these genes are key negative regulators that not only increase aggressiveness in pancreatic cancer cells but also contribute to ferroptosis resistance. Moreover, we showed that correlations between expressions of FTH1 and the ferroptosis regulators, *FDFT1* and *LPCAT3*, may be linked to initiation of pancreatic tumors (Table 2). *FDFT1* encodes squalene synthase (SQS) as a target protein of FIN56, which promotes degradation of the lipid repair enzyme, *GPX4*, and induces accumulation of lipid peroxidation^51^. *LPCAT3* is the gene that is involved in phospholipid synthesis and induces ROS accumulation to drive ferroptotic cell death^17^. Collectively, our analysis of TCGA dataset suggested that several candidate genes may be associated with the onset and progression of pancreatic cancer.

There are several limitations of our study. First, there were only 68 Taiwanese participants used for the analysis, which may lead to potential type II errors^52^. To reduce the risk of false-negative results, we conducted a meta-analysis which included 315 subjects from six eligible articles, to overcome the limitation of a small sample size in our case–control study. Second, methodological variations among the included studies may have led to statistical heterogeneity. Five articles measured ferritin concentration by IRMA or 2-site IRMA. whereas one study conducted by Hamazoe et al. measured SF by RIA, which may have led to a sensitivity difference between the assays^53,54^. In addition, there was a lack of obstructive jaundice information of PDAC patients. Previous studies emphasized that the presence of jaundice improves the biomarker specificity for PDAC patients^55,56^. Considering jaundice as a possible confounding factor for PDAC biomarker discovery, further studies are required to examine whether the presence of obstructive jaundice in PDAC patients may influence the level of serum ferritin^55^.

However, despite these limitations, our study is the first population-based case–control study that presented SF levels of PDAC patients and healthy controls in Taiwan, including a pooled-analysis of six studies to further strengthens the evidence present on the association between SF and pancreatic cancer. The present results are consistent with a recent meta-analysis study from Lin et al., which showed an elevated pretreatment SF is associated with poorer survival outcomes in hepatobiliary and pancreas (HBP) cancer patients, emphasizing the possibility of utilizing SF as the prognostic value for pancreatic cancer^57^. We also aimed to update a previous finding of related topic with the inclusion of new cohort study, since former six studies that have compared SF levels in pancreatic cancer patients and controls were published in either late 1980s or early 1990s. Further to this, we utilized Oncomine database as a tool to identify several genes that modulate ferritin and predict a poorer prognosis of pancreatic cancer, presenting new possibilities for research and ultimate utilization for cancer therapies. More studies, however, are needed to elucidate the regulation of ferritin in pancreatic tumors.

## Methods

### Ethics declarations

The study was conducted in accordance with the Declaration of Helsinki. The tissues were obtained from Taipei Medical University Joint Biobank and study was conducted according to the ethical approval by the Ethics Committee of Taipei Medical University (Approval number N202001052).

### Search strategy and selection criteria for the meta-analysis

A search of PUBMED and EMBASE was conducted by two investigators who reviewed studies published up to 1 March 2020. The search was restricted to the English and Chinese languages and human subjects, but one article written in Japanese was later included from a hand search. The search keywords included: “serum ferritin” OR “ferritin” AND “pancreatic cancer”. Search questions were based on the PICO framework: 1. an original research article published in a peer-reviewed journal; 2. the population included adults with pancreatic cancer who were aged ≥ 18 years; 3. the intervention was the serum level of ferritin; 4. the comparison intervention was non-pancreatic cancer subjects, controls or healthy subjects; and 5. the intervention outcome included pancreatic cancer occurrence. Retrieved records were sent to Endnote© (vers. 7.3.1; © 2019 Clarivate) and duplicates were removed. Articles were excluded if they did not meet the predefined inclusion criteria, did not include human subjects, were a review or case report, and did not provide SF levels for both pancreatic cancer subjects and healthy controls. We acknowledge that two publications included in the meta-analysis were from the same authors^19,22^, but the number of subjects and the measured SF levels in both healthy controls and pancreatic cancer patients differ between the two studies; thus, we included these publications as two separate studies.

### Quality assessment for individual studies

Study quality was evaluated by two independent researchers (Ji Min Park and Chen-Zou Mau), using the Effective Public Practice Project (EPHPP) Quality Assessment Tool^58,59^. Each study was rated as being weak (1 point), moderate (2 points), or strong (3 points) for risk of bias within six domains: study bias, study design, confounders, blinding, data collection methods, and withdrawals and drop-outs. In the case of discrepancies, discussion and a detailed examination of the full text was performed (Supplementary Table S1). The total score of the final decision was achieved after averaging domain scores.

### Data analysis

A meta-analysis was conducted using R Studio 3.5.3. (Boston, MA, USA)^33^. We estimated a pooled standard mean difference (SMD) and the 95% confidence interval (CI) on the basis of both fixed-effect and random-effect models. The standard deviation (SD) was calculated as the standard error (SE) using the equation: SD = SE * $\sqrt n$ (*n* = population size). Between-study heterogeneity was calculated using the Cochrane Q-test, and values were evaluated as tau^2^, Chi^2^, and I^2^, where a higher I^2^ value indicates high heterogeneity between studies. Generally, I^2^ values of 25%, 50%, and 75% respectively indicate low, moderate, and high heterogeneity. Publication bias was examined using a funnel plot and Egger’s test. Further, we evaluated the potential sources of heterogeneity by fitting linear meta-regression models^60^.

### Sample collection and patients

Preoperative serum samples were obtained from Taipei Medical University Joint Biobank, including 34 Taiwanese patients with PDAC and 34 age-matched and gender-matched healthy controls, whose age ranged 41–82 years. Tumor grades and stages were reviewed by senior gastrointestinal pathologists according to the 7th edition of American Joint Committee on Cancer/International Cancer Control (AJCC/UICC). Clinical data on gender, age, tumor location, TNM tumor stage, histologic type and grade, and pathological features were obtained from the TMU Joint Biobank. Five patients (14.7%) were in grade 1 (G1), 20 patients (58.8%) were in grade 2 (G2), and nine patients (26.5%) were in grade 3 (G3). No patients were in histological grade 4 (G4). Briefly, a 10-mL sample of peripheral blood was collected from PDAC patients and healthy controls in a tube that contained separating gel and clot activator. Test tubes were centrifuged at 3400 rpm for 7 min, and serum was aliquoted after the clot was removed. Serum samples were then stored at − 80 °C until being analyzed. SF was assessed by an electrochemiluminescence immunoassay (ECLIA) and was quantified with a Roche Modular P800 analyzer (Roche Diagnostics, Mannheim, Germany).

### Gene set analysis

A gene set analysis was performed using R Studio 3.5.3^33^ using the packages meta^61^ (v4.9.4) and metafor^62^ (v2.0.0) on the dataset from TCGA cohorts and the Oncomine database. In total, 10 normal pancreatic tissue samples and 51 pancreatic tumor tissues were included for the gene set analysis; tumor tissues were then sub-grouped into grade 1 (G1), grade 2 (G2), or grade 3 (G3) according to histological diagnoses. Differential expression was calculated based on z-scores showing up regulated (red) and down regulated (green) genes clustered in heatmaps. Statistical analyses were performed with GraphPad prism 8.4.1. Gene sets associated with histologic tumor grades of pancreatic cancer were statistically evaluated using a two-way analysis of variance (ANOVA) with Tukey’s post-hoc tests.

### Statistical methods

All analyses were performed using GraphPad prism 8.4.1. A *p* value of < 0.05 was considered to indicate significance. Significance in all figures is indicated as follows: *p < 0.05, **p < 0.01, ***p < 0.001, and ****p < 0.0001.

## Supplementary Information

Supplementary Information.
