# Supplementary material for: A case–control study in Taiwanese cohort and meta-analysis of serum ferritin in pancreatic cancer
Source: Sci Rep. 2021 Oct 28;11:21242. doi: 10.1038/s41598-021-00650-7 (PMC8553768; doi:10.1038/s41598-021-00650-7)
Supplement: Supplementary file 1 — Supplementary Information. [file 41598_2021_650_MOESM1_ESM.docx]

**Table S1. Quality assessment of the six included studies using the Effective Public Health Practice Project tool**

| **Study** | **Selection bias** | **Study design** | **Confounders** | **Blinding** | **Data collection methods** | **Withdrawals and dropouts** | **Global score** |
| --- | --- | --- | --- | --- | --- | --- | --- |
| Nitti 1982 [33] | Strong | Moderate | Weak | Moderate | Strong | Not applicable | Moderate |
| Fabris 1984 [18] | Strong | Moderate | Weak | Moderate | Strong | Not applicable | Moderate |
| Hamazoe 1984 [34] | Strong | Strong | Weak | Moderate | Strong | Not applicable | Moderate |
| Chen 1984 [24] | Strong | Moderate | Weak | Moderate | Strong | Not applicable | Moderate |
| Fabris 1985 [19] | Strong | Moderate | Weak | Moderate | Strong | Not applicable | Moderate |
| Basso 1991 [35] | Moderate | Strong | Weak | Moderate | Strong | Not applicable | Moderate |

**Table S2. Univariate meta-regression analysis of six eligible studies**

| **Variable** | **SE** | **Z value** | ***p*-value** | **95% CI** | **I^2^ (%)** |
| --- | --- | --- | --- | --- | --- |
| **Measurement** |  |  |  |  |  |
| IRMA | 2.091 | 0.102 | 0.918 | (-2.076 to 6.665) | 95.3 |
| RIA | 2.230 | 1.029 | 0.303 | (-3.883 to 4.312) |  |
| **Region** |  |  |  |  |  |
| Europe | 1.366 | -1.539 | 0.124 | (-4.779 to 0.575) | 94.1 |
| **Publication** | 0.279 | -0.406 | 0.685 | (-0.659 to 0.433) | 96.1 |
| **Control** |  |  |  |  |  |
| Number of controls | 0.008 | 7.084 | <0.001 | (0.040 to 0.071) | 0.00 |
| % of women | 0.030 | 0.179 | 0.858 | (-0.054 to 0.065) | 0.00 |
| **Patients** |  |  |  |  |  |
| Number of patients | 0.051 | -3.733 | <0.001 | (-0.291 to -0.091) | 82.2 |
| % of women | 0.012 | -0.656 | 0.512 | (-0.033 to 0.016) | 0.00 |

CI, Confidence intervals; IRMA, Immunoradiometric assay

**Table S3. Univariate meta-regression analysis of five eligible studies (exclusion of a study by Chen *et al.*)**

| **Variable** | **SE** | **Z value** | ***p*-value** | **95% CI** | **I^2^ (%)** |
| --- | --- | --- | --- | --- | --- |
| **Measurement** |  |  |  |  |  |
| IRMA | 0.387 | 0.549 | 0.583 | (-0.546 to 0.970) | 0.00 |
| RIA | 0.457 | 0.229 | 0.819 | (-0.790 to 0.999) |  |
| **Region** |  |  |  |  |  |
| Europe | 0.367 | 0.12 | 0.904 | (-0.676 to 0.764) | 0.00 |
| **Publication** | 0.049 | -0.489 | 0.625 | (-0.119 to 0.071) | 0.00 |
| **Control** |  |  |  |  |  |
| Number of controls | 0.131 | -0.423 | 0.672 | (-0.311 to 0.201) | 0.00 |
| % of women | 0.030 | 0.179 | 0.858 | (-0.054 to 0.065) | 0.00 |
| **Patients** |  |  |  |  |  |
| Number of patients | 0.040 | -0.405 | 0.685 | (-0.095 to -0.062) | 0.00 |
| % of women | 0.012 | -0.656 | 0.512 | (-0.033 to 0.016) | 0.00 |

CI, Confidence intervals; IRMA, Immunoradiometric assay

**Table S4. Serum ferritin of 68 participants measured by an electrochemiluminescence assay (ECLIA) including pancreatic ductal adenocarcinoma (PDAC) patients (n = 34) and healthy controls (n = 34)**

| **Measurement** | **PDAC** | | | | **Healthy controls** | | | |
| --- | --- | --- | --- | --- | --- | --- | --- | --- |
|  | **Median age (IQR) (yr)** | **N** | **% of women** | **Concentration**  **(mean** $\boldsymbol{\pm}$**SD)** | **Median age (IQR) (yr)** | **N** | **% of women** | **Concentration**  **(mean** $\boldsymbol{\pm SD}$**)** |
| ECLIA | 66.0  (58.0-71.0) | 34 | 47 | 608.4$\pm736$.3 ng/ml | 62.0  (59.8-63.0) | 34 | 47 | 190.7$\pm163$.8 ng/ml |

ECLIA, Electrochemiluminescence assay; PDAC, pancreatic ductal adenocarcinoma

**Table S5. Table of serum ferritin for subjects with healthy controls and pancreatic ductal adenocarcinoma patients classified according to their histological grades or tumor stages**

| **Participants** | **Healthy controls** | **PDAC patients (n = 34)** | | | | |
| --- | --- | --- | --- | --- | --- | --- |
|  |  | **Histological grades** | | | **Tumor stages** | |
|  |  | **G1** | **G2** | **G3** | **Stage**  **0-IIB** | **Stage**  **III-IV** |
| **N (%)** | **34** | 5 (14.7) | 20 (58.8) | 9 (26.5) | 30 (88.2) | 4 (11.8) |
| **Age Median**  **(95% CI)** | 62.00  (60.51-62.87) | 67.00  (43.39-79.41) | 62.00  (55.37-67.57) | 65.00  (62.02-73.09) | 67.50  (61.63-69.37) | 62.50  (57.97-68.03) |
| **Concentration**  **(mean**$\boldsymbol{\pm}$**SD), ng/ml** | 190.7 $\pm$ 163.8 | 405.3 $\pm$ 356.7 | 769.3 $\pm908.7$ | 363.6$\pm$ 232.1 | 662.6 $\pm$ 757.3 | 201.8 $\pm$ 152.5 |
| ***p*-value** |  | 0.8271 | 0.0012 | 0.8144 | 0.0019 | 0.9991 |

*p*-value indicates the statistical significance of serum ferritin concentration compared to control, as determined by one-way ANOVA with Tukey’s post-hoc tests.


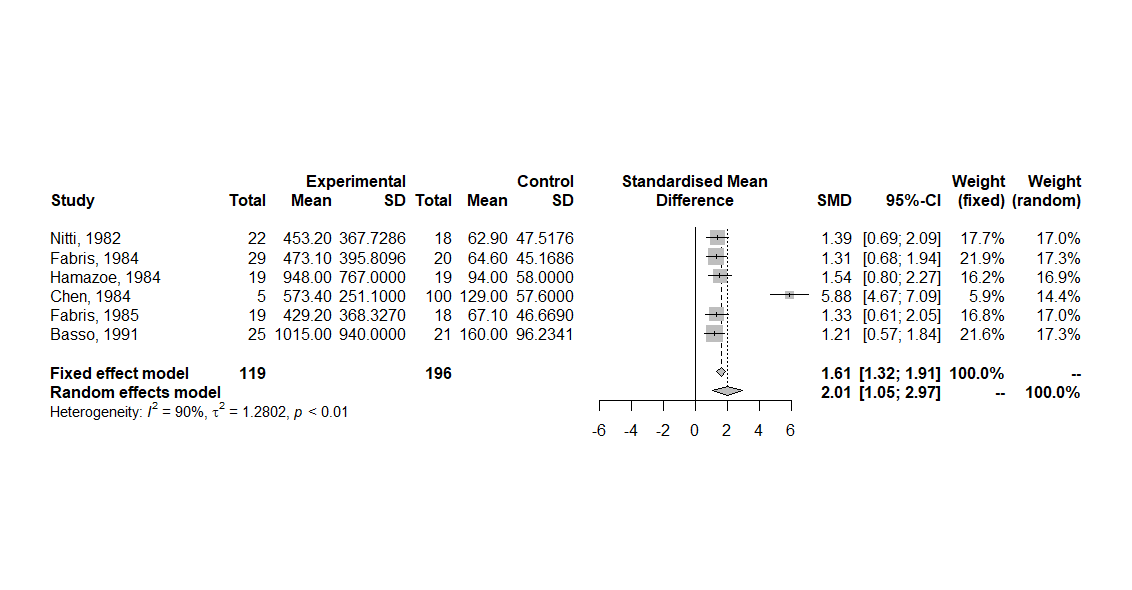


**Figure S1.** Forest plot of studies in serum ferritin (SF) for subjects with pancreatic cancer (Experimental; n = 119) versus healthy controls (Control; n = 196). Mean difference (MD) and 95% confidence intervals (CI) were calculated using both fixed and random effect models.

**
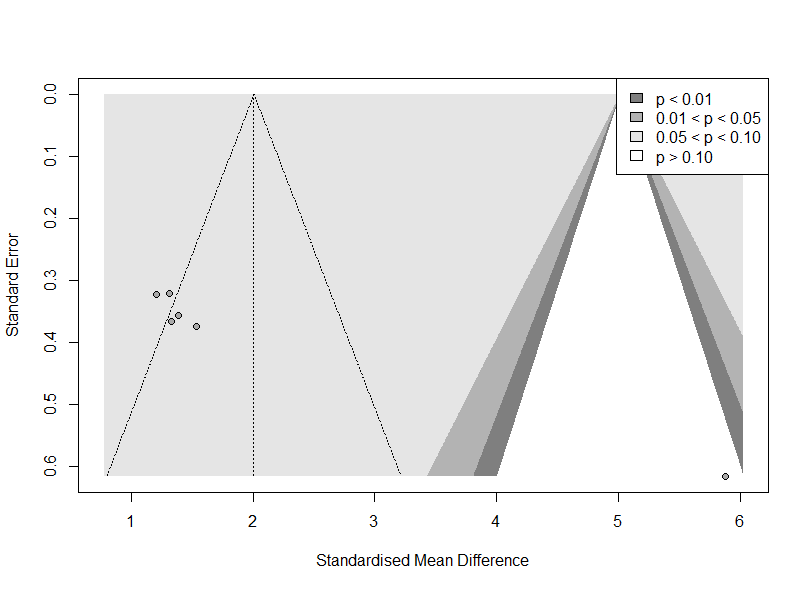
**

**Figure S2.** Funnel plot of studies in serum ferritin (SF) for subjects with pancreatic cancer versus healthy control.
